# Integrative Medicine (Herbal Medicine Combined with Drug Therapy) for Behcet’s Disease: A Systematic Review and Meta-Analysis of Randomized Controlled Trials

**DOI:** 10.3390/pharmaceutics13040476

**Published:** 2021-04-01

**Authors:** Ji Hee Jun, Lin Ang, Tae Young Choi, Hye Won Lee, Myeong Soo Lee

**Affiliations:** 1 Clinical Medicine Division, Korea Institute of Oriental Medicine, 1672 Yuseongdae-ro, Yuseong-gu, Daejeon 34054, Korea; zhixi04@kiom.re.kr (J.H.J.); anglin2808@kiom.re.kr (L.A.); superoung@kiom.re.kr (T.Y.C.); 2 Korean Convergence Medicine, University of Science and Technology, Daejeon 34113, Korea; 3 Herbal Medicine Research Division, Korea Institute of Oriental Medicine, Daejeon 34054, Korea; hwlee@kiom.re.kr

**Keywords:** integrative medicine, complementary and alternative medicine, herbal medicine, Behcet’s disease, systematic review

## Abstract

This review aimed to investigate the efficacy of integrative medicine (herbal medicine combined with drug therapy) in the treatment of Behcet’s disease (BD). Eleven databases were searched from their inception to 7 December 2020, for randomized control trials (RCTs) that reported the effects of integrative medicine in treating BD. The risk of bias was assessed using seven domain criteria from the Cochrane Collaboration tool. Grading of Recommendations Assessment, Development, and Evaluation (GRADE) was used to assess the quality of evidence. The direction of the effect is also shown in the form of an albatross plot. Sixteen trials met the inclusion criteria and were analyzed. The overall risk of bias was determined to be uncertain. The meta-analysis showed a superior response rate with herbal medicine plus drug therapy (relative risk (RR) 1.19, 95% confidence interval (CI) 1.13 to 1.25, *n* = 1034, *p* < 0.00001, I^2^ = 0%, low certainty of evidence (CoE)) compared to drug therapy. Integrative medicine also lowered the recurrence rate after 2 months of follow-up (RR 0.27, 95% CI 0.09 to 0.76, *n* = 120, *p* = 0.01, I^2^ = 0%, low CoE). The erythrocyte sedimentation rate (ESR), C-reactive protein (CRP) level, and skin lesions were also significantly improved using integrative medicine, but equivalent effects were seen for oral ulcers, genital ulcers, and eye inflammation. Minor adverse events were reported in both groups. Our findings suggest that herbal medicine combined with drug therapy is more effective for the treatment of BD than drug therapy alone. Although the type of drug therapy used varied across the studies, integrative medicine was shown to improve the total response rate, skin lesions, the ESR, and the CRP level. However, the overall risk of bias of the studies was concerning, and the CoE was low. Information on adverse events (AEs) was also insufficient. In addition, the number of studies included for data synthesis for most outcomes was small. Future studies with rigorous RCTs may help establish the efficacy of integrative medicine in the treatment of BD.

## 1. Introduction

Behcet’s disease (BD) is a multisystemic inflammatory disorder characterized by a range of manifestations, such as recurrent oral ulcers, genital ulcers, arthritis, vasculitis, and skin lesions. The age of onset of BD is usually 30–40 years [1,2]. The prevalence of BD is higher in the Middle East and Asia than in North America and northern Europe. In particular, its prevalence is highest in Turkey (80–370 cases per 100,000 persons) [3,4,5,6]. BD is commonly treated with steroids and immunomodulatory drugs such as corticosteroids, colchicine, and thalidomide, which often present side effects such as peripheral neuropathy, loss of appetite, nausea, diarrhea, and intestinal bleeding or perforation [7,8,9].

Integrative medicine refers to the combination of conventional drug therapies with complementary or alternative medicine (CAM) therapies such as acupuncture and herbal medicine [10]. Integrative medicine combines the benefits of both Western medicine and CAM [11]. It has, therefore, become increasingly prevalent and popular, not only in China but also worldwide [12]. Clinical trials have revealed that a combination of herbal medicine and drug therapy is more effective than herbal medicine alone and significantly reduces the symptoms and recurrence rate of BD. This improvement is accompanied by the expression of the cytokine LI-4 [13,14]. A recent publication also reported that herbal medicine was favorable for treating BD, showing the potential of herbal medicine in complementing conventional medication [15].

Two systematic reviews (SRs) have been published on the use of integrative medicine for the treatment of BD [16,17]. These SRs included seven randomized controlled trials (RCTs) that compared the effects of integrative medicine with drug therapy; however, the included studies did not have publication dates beyond 2014. Thus, this study aimed to update the evidence on the efficacy of integrative medicine in the treatment of BD.

## 2. Methods

### 2.1. Study Registration and Protocol Information

This review has been registered on PROSPERO CRD4201808496.

### 2.2. Data Sources

Eleven electronic databases, namely, PubMed, Embase, Cochrane Controlled Register of Trials (CENTRAL), China National Knowledge Infrastructure (CNKI), Wanfang Data, VIP Information, OASIS, DBpia, Research Information Service System (RISS), Korean Studies Information Service System (KISS), and KoreaMed, were searched from inception to 7 December 2020. Our search included studies in English, Chinese, and Korean languages. The search strategy included Medical Subject Headings (MeSH) and keywords that reflected the terms related to Behcet’s syndrome, BD, Behçet’s, integrative medicine, and integrated medicine. Detailed search terms are shown in Supplementary Materials.

### 2.3. Study Selection

#### 2.3.1. Types of Studies

Eligible studies included RCTs or quasi-RCTs that compared integrative medicine with drug therapy. Postgraduate theses or dissertations were also eligible. Publications in the form of abstracts, conference proceedings, review articles, or other types of clinical studies, such as nonrandomized controlled studies, case-control studies, case reports and series, and animal studies, were not eligible.

#### 2.3.2. Types of Participants

Eligible participants were patients of both sexes and all ages clinically diagnosed with BD. The studies had to meet the following diagnostic criteria for inclusion:

- International Study Group (ISG) criteria [18];
- International Criteria for BD (ICBD) criteria [18];
- Criteria of diagnosis and therapeutic effect of diseases and syndromes in traditional Chinese medicine [19];
- Guidelines for the diagnosis and treatment of BD by the Chinese Rheumatology Association (CRA) [20]; and
- Guiding principle of clinical research on new drugs of traditional Chinese medicine [21].

#### 2.3.3. Types of Interventions and Comparison

Studies where patients received herbal medicine combined with drug therapy as an intervention were eligible. Concerning herbal medicine, we included only those studies that used herbal decoctions. There were no limitations on the number, administration methods, dosage, or duration of treatment. Studies that integrated other types of CAM therapies, such as moxibustion, acupuncture, massage, and cupping with drug therapy, were excluded.

Studies that used drug therapy alone, no treatment, or placebo as comparators were eligible. Studies in which the comparators included other types of herbal medicine were excluded.

#### 2.3.4. Types of Outcome Measurements

Primary Outcomes

- Total relative risk (RR): (recovery + marked improvement + improvement)/total number of cases × 100%.
- Recurrence rate.

Secondary Outcomes

- Laboratory changes in C-reactive protein (CRP) levels and the erythrocyte sedimentation rate (ESR);
- Symptom score (oral ulcers, genital ulcers, eye inflammation, skin lesions, and arthralgia); and
- Adverse events (AEs).

### 2.4. Data Extraction and Risk of Bias Assessment

#### 2.4.1. Data Extraction

We searched and screened eleven databases by checking the title and abstract. Furthermore, we independently extracted detailed data from the studies pertaining to the seven following domains: (1) first author, year of publication; (2) diagnosis, sample size, duration of treatment; (3) intervention group; (4) control group; (5) main outcomes; (6) results; and (7) AEs. In addition, we obtained the details on the prescription of herbal treatment.

#### 2.4.2. Risk of Bias

As an assessment of the quality of each study, two authors (J.H.J and T.Y.C) used Cochrane Collaboration’s tool to evaluate the risk of bias [22]. We evaluated seven domains, namely, random sequence generation, allocation concealment, blinding of participants and personnel, blinding of the outcome assessment, incomplete outcome of the data, selective reporting, and other biases, to assess the risk of bias. We categorized the risk of bias as low (L), high (H), or uncertain (U). Disagreements were resolved by another author (MSL).

#### 2.4.3. Data Analysis

Data analyses were performed using Review Manager (Version 5.3.5) software provided by the Cochrane Collaboration. We quantified the effects of treatment as the RR with the 95% confidence interval (CI) for dichotomous data and as the mean difference (MD) with the 95% CI for continuous data. The chi-square test and Higgins I^2^ test were used to assess heterogeneity. Additionally, we used Grading of Recommendations Assessment, Development, and Evaluation (GRADE) pro/GDT to assess the certainty of evidence (CoE) (21). Albatross plots were also generated using STATA/SE v.16.1 (StataCorp LLC, College Station, TX, USA) to visualize the effect of direction on the primary and secondary outcomes.

## 3. Results

### 3.1. Descriptions of the Included Trials

**Figure 1:**
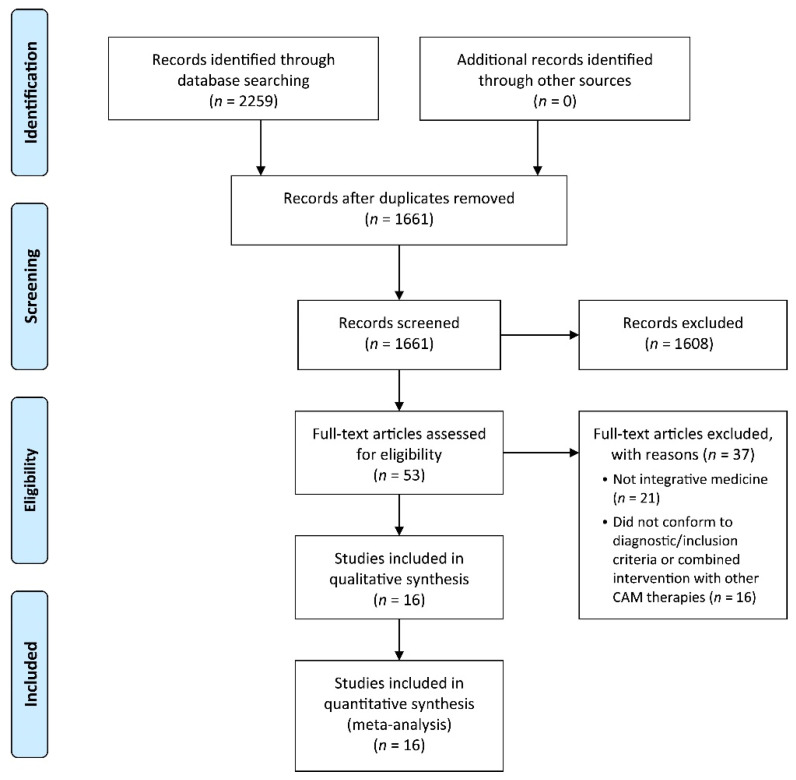
Flow chart of the study selection process.

After the literature search, 2259 records were retrieved, and 1661 titles and abstracts were screened after removing duplicates. Fifty-three eligible articles were assessed, and 37 articles were eliminated for various reasons, leaving 16 full-text articles [23,24,25,26,27,28,29,30,31,32,33,34,35,36,37,38] that met the inclusion criteria for final inclusion (Figure 1). All included studies were conducted in China and published between 2002 and 2020 in the Chinese language.

All studies were two-arm parallel-designed trials. The mean age of patients from the included studies ranged from 23.4 to 41.3 years. The average disease duration ranged from 2.5 to 12.6 years. The total sample size was 987, with the number of participants in each group ranging from 20 to 57. All included studies used integrative medicine (combination of herbal medicine and drug therapy) as the intervention and drug therapy alone as the comparator. The details of the included studies are shown in Table 1.

Herbal medicine interventions in the included studies were herbal prescriptions given in the form of oral decoctions. Thirteen studies used standard prescriptions or modified prescriptions, whereas the remaining three studies used pattern identification (PI)-based prescriptions. The standard and modified prescriptions included modified Gancao Xiexin decoction, modified Qingdai san, Shen’s Shengdi Qinlian Tufuling decoction, modified Huanglian decoction, modified Yiqi Jiedu Quyu prescription, standard/modified Huiyan Zhuyu decoction, Baitouweng decoction, Yinshenhao decoction, Duanxia Shenshi decoction, Longdan Xiegan decoction, modified Chixiaodou Danggui San, modified Ziyin Yuyang decoction, and Leiling Jiedu decoction. The herb with the highest frequency of use across the prescriptions was Glycyrrhizae Radix et Rhizoma, followed by Scutellariae Radix and Coptidis Rhizoma. The herbal compositions of the included prescriptions are presented in Table 2. The conventional drugs included thalidomide, celecoxib, azathioprine, prednisone, levamisole, and cyclophosphamide (all administered orally), and dexamethasone (administered intravenously).

### 3.2. Risk of Bias

**Figure 2:**
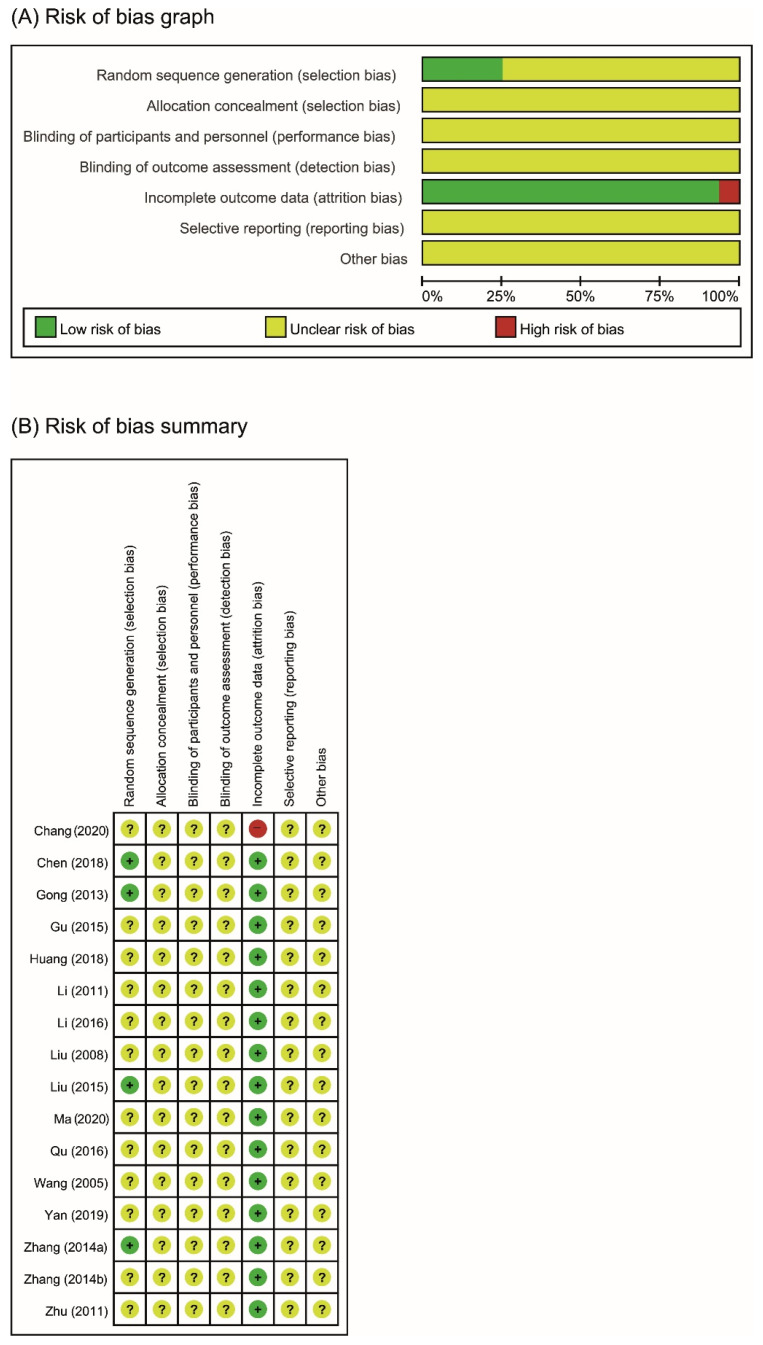
Risk of bias. (**A**) Risk of bias is shown as a graph: review authors’ judgments on each item’s risk of bias are presented as percentages across all included studies. (**B**) Risk of bias summary: review authors’ judgments on each item’s risk of bias for each included study. +: low risk of bias; −: high risk of bias; ?: unclear risk of bias.

The overall risk of bias was judged to be uncertain using Cochrane Collaboration’s tool for risk of bias assessment (Figure 2).

Only four of the included studies reported the method used for random sequence generation, and none of the studies used allocation concealment methods [23,24,25,28]. As none of the studies reported the blinding of participants/personnel or outcome measurements, performance biases and detection biases for all studies were judged as unclear. Most studies were judged as having a low risk of bias because follow-up information or outcome data were missing; however, one study was judged as having a high risk of bias due to the incomplete report of several outcomes [37]. One study (27) that reported a few dropouts was eventually judged as having a low risk of bias, as the number of dropouts was less than 10% of the randomized samples, and the remaining studies did not report the percentage of patients who were lost to follow-up or did not perform an intention-to-treat (ITT) analysis [28]. The judgment of unclear risk of bias was given to all studies for reporting bias, as none of the studies had study protocols and did not provide sufficient information for further assessment. Information such as the source of funding, sample size calculation, and trial registration was also insufficient to assess other potential biases in the included studies.

### 3.3. Certainty of Evidence

The CoE for each outcome as assessed using GRADE was low or very low. The ‘Summary of findings’ table on the main outcomes is presented in Table 3.

### 3.4. Outcome Measurements

#### 3.4.1. Primary Outcomes

##### Total Response Rate

**Figure 3:**
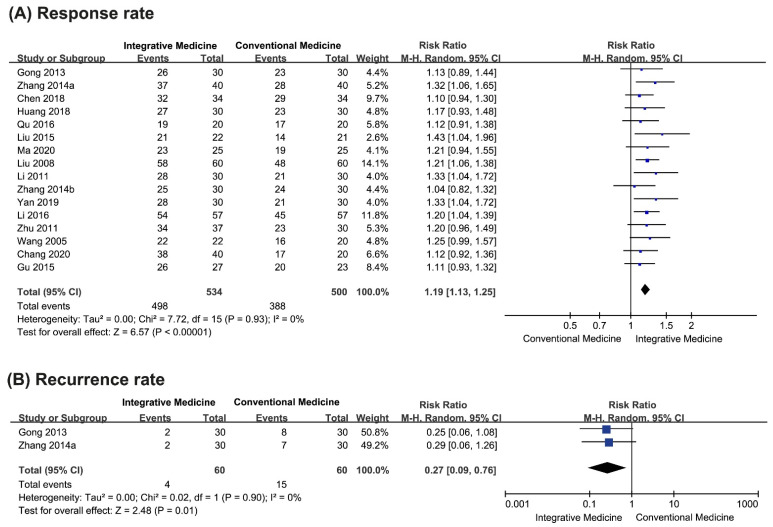
Forest plots of primary outcomes. (**A**) Total response rate; (**B**) recurrence rate.

Sixteen studies assessed the total response rate. Six studies reported a superior effect of integrative medicine compared with drug therapy [23,24,25,26,27,28,29,30,31,32,33,34,35,36,37,38], while the other ten studies reported equivalent effects between the two groups. The meta-analysis showed a favorable effect towards integrative medicine (RR 1.19, 95% CI 1.13 to 1.25, *n* = 1034, *p* < 0.00001, I^2^ = 0%, low CoE, Figure 3A).

##### Recurrence Rate

Only two studies that compared the effectiveness of herbal medicine combined with drug therapy with drug therapy assessed the recurrence rate [23,24]. One study [23] reported the recurrence rates between the two groups after 1 month, 2 months, and 3 months of follow-up. The recurrence rate was equivalent between the two groups after 1 month (RR 0.33, 95% CI 0.04 to 3.03, *p* = 0.33) and 2 months (RR 0.25, 95% CI 0.06 to 1.08, *p* = 0.06) of follow-up. After 3 months of follow-up, the recurrence rate was lower for the herbal medicine combined with drug therapy group than for the drug therapy group (RR 0.27, 95% CI 0.08 to 0.88, *p* = 0.03). Another study [24] reported the recurrence rate only at the 2-month follow-up (RR 0.25, 95% CI 0.06 to 1.08, *p* = 0.10).

A meta-analysis of both studies on the recurrence rate after 2 months of follow-up showed that the group administered herbal medicine combined with drug therapy experienced a lower recurrence rate than the group administered drug therapy alone (RR 0.27, 95% CI 0.09 to 0.76, *n* = 120, *p* = 0.01, I^2^ = 0%, low CoE, Figure 3B).

#### 3.4.2. Secondary Outcomes

##### Symptom Score

*Oral Ulcers*

**Figure 4:**
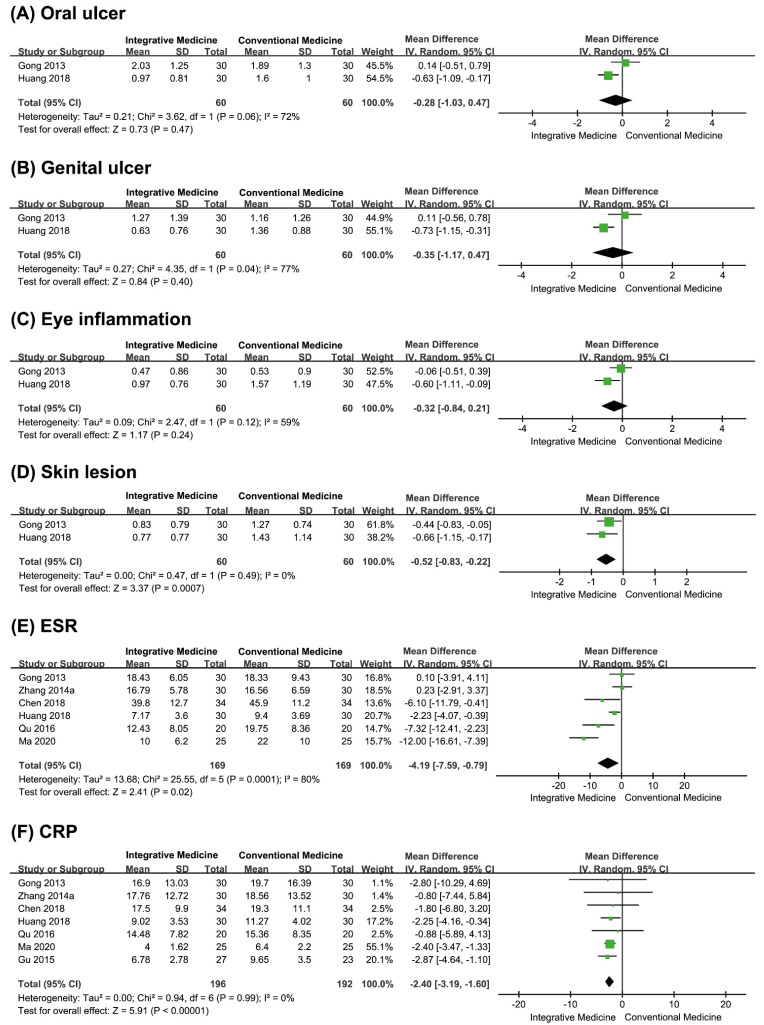
Forest plots of secondary outcomes. (**A**) Oral ulcers, (**B**) genital ulcers, (**C**) eye inflammation, (**D**) skin lesions, (**E**) ESR, (**F**) CRP.

Only two studies reported the symptom score for oral ulcers [23,26]. The meta-analysis showed an equivalent effect between the herbal medicine combined with drug therapy group and the drug therapy group (MD −0.28, 95% CI −1.03 to 0.47, two studies, *n* = 120, *p* = 0.47, I^2^ = 72%, very low CoE, Figure 4A).

*Genital Ulcers*

Only two studies assessed the symptom score for genital ulcers [23,26]. The meta-analysis showed an equivalent effect between the herbal medicine combined with drug therapy group and the drug therapy group (MD −0.35, 95% CI −1.17 to 0.47, *n* = 120, *p* = 0.40, I^2^ = 77%, very low CoE, Figure 4B).

*Eye Inflammation*

Only two studies evaluated the symptom score for eye inflammation [23,26]. The meta-analysis showed an equivalent effect between the herbal medicine combined with drug therapy group and the drug therapy group (MD −0.32, 95% CI −0.84 to 0.21, *n* = 120, *p* = 0.24, I^2^ = 59%, very low CoE, Figure 4C).

*Erythrocyte Sedimentation Rate and C-Reactive Protein Levels*

Four studies showed a favorable effect of herbal medicine combined with drug therapy compared to drug therapy alone [25,26,27,29], while two studies showed an equivalent effect between the two groups [23,24]. Of the six studies [23,24,25,26,27,29], one was excluded from the meta-analysis due to incomplete reporting [37]. The meta-analysis showed that the effect of herbal medicine combined with drug therapy on the ESR was superior to that of drug therapy alone (MD −4.19, 95% CI −7.59 to −0.79, *n* = 338, *p* = 0.02, I^2^ = 80%, very low CoE, Figure 4E).

Four studies showed a favorable effect of herbal medicine combined with drug therapy compared to drug therapy alone [26,30,37,38], while four other studies showed an equivalent effect between the two groups [23,24,25,27]. Among the eight studies [23,24,25,26,27,30,37,38], one was excluded from the meta-analysis due to incomplete reporting [37]. The meta-analysis showed that herbal medicine combined with drug therapy had a superior effect on CRP levels (MD −2.40, 95% CI −3.19 to −1.60, *n* = 388, *p* < 0.0001, I^2^ = 0%, low CoE, Figure 4F) compared to drug therapy alone.

Adverse events

Nine studies assessed AEs [23,24,25,26,27,29,36,37,38]. Of the nine studies, two [26,36] reported no AEs for either integrative therapy or drug therapy. In the remaining seven studies, 30 AEs were reported in the herbal medicine combined with drug therapy group, and 67 AEs were reported in the drug therapy alone group. The details of the AEs are listed in Table 1.

### 3.5. Albatross Plot

**Figure 5:**
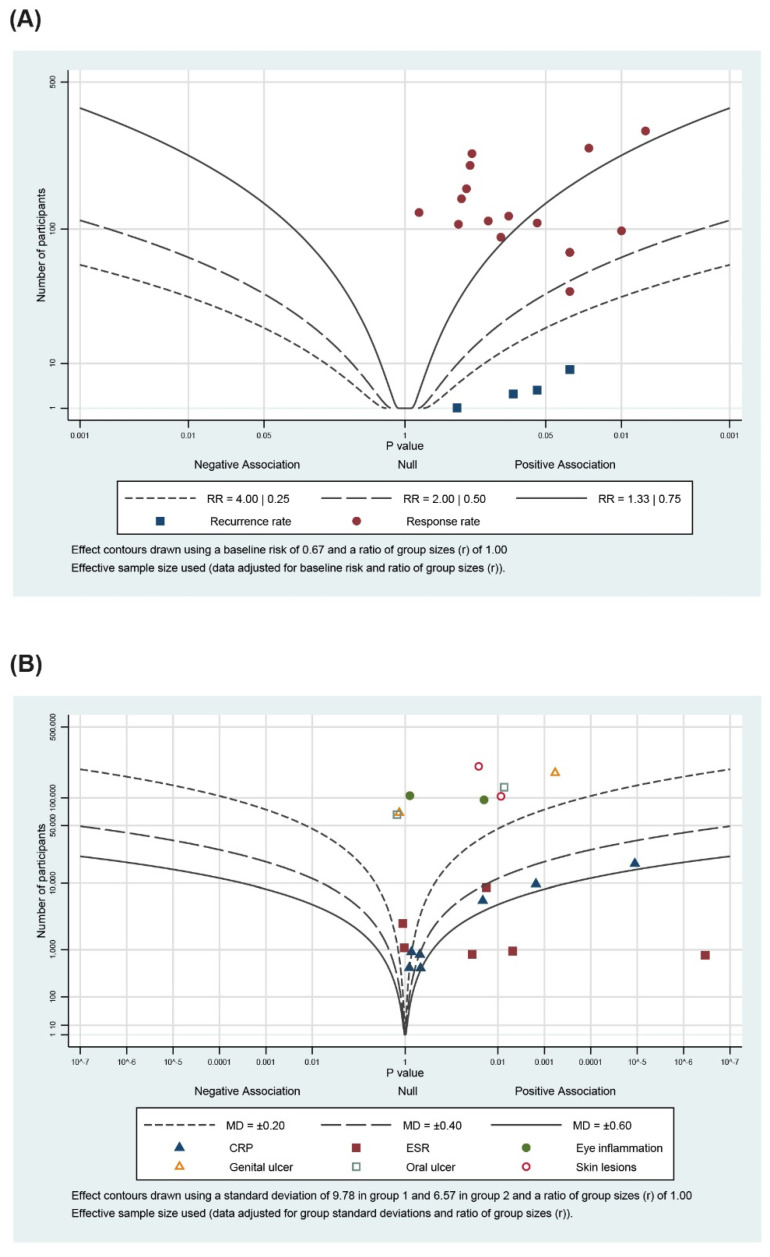
Albatross plot for (**A**) primary outcomes and (**B**) secondary outcomes.

The albatross plot showing the effects of direction and size range by *p*-value and the given sample sizes was generated for each included study (Figure 5, different outcome groups are presented in different colors). For the dichotomous data, the points were scattered across the contour lines (Figure 5A). All the points were clustered to the positive association side of the plot, implying that herbal medicine combined with drug therapy is favorable for the treatment of BD. For the continuous data, the points were scattered more towards the right side of the plot, with many points clustered around the null line, showing the equivalent effect of herbal medicine combined with drug therapy and drug therapy alone (Figure 5B).

## 4. Discussion

### 4.1. Summary of the Main Results

This systematic review revealed that herbal medicine combined with drug therapy appears to be more effective for the treatment of BD than drug therapy alone. Although the type of drug therapy used varied across the studies, herbal medicine combined with drug therapy was shown to improve the total response rate, skin lesions, ESR, and CRP level. However, the overall risk of bias of the studies was concerning, and the CoE was low. Information on AEs was also insufficient. In addition, the number of studies included for data synthesis for most outcomes was small. Future studies with rigorous RCTs may help establish the efficacy of herbal medicine combined with drug therapy in the treatment of BD.

### 4.2. Quality of the Evidence

The level of evidence as assessed by GRADE for the studies included in this meta-analysis was low. All the included studies had a high risk of bias. The evidence was downgraded by one level in the category of risk of bias due to a lack of information on the randomization, allocation concealment, and blinding methods in all studies. Further high heterogeneity of the symptom score outcome (oral ulcers, genital ulcers, and eye inflammation) downgraded the quality of evidence by one level in the category of inconsistency. In the category of indirectness, the included studies corresponded to the patient, intervention, comparison, outcomes (PICO) study, and it was ascertained that this effect was not serious. In the category of imprecision, the included studies had a small sample size, with 10 to 57 participants; thus, the quality of evidence was downgraded by one level. In general, the quality of reporting was poor in the included trials. Furthermore, the number of trials and total sample size included in our analysis were not sufficient to draw firm conclusions.

### 4.3. Potential Biases in the Review Process

There were several limitations to the present review. First, all the studies were published in China, limiting the overall generalization of the results. Despite searching eleven databases, all the studies that met the inclusion criteria were performed in China, where no studies with negative results have been reported [39]. Second, the methodological quality of the included studies was poor. There was a lack of information on randomization procedures and blinding methods that led to a concerning risk of bias. Third, the numbers of studies included in the meta-analyses of several outcomes, such as major BD symptoms and the recurrence rate, were small, resulting in a lack of significance and inconclusive results. In addition, the heterogeneity of herbal medicines used in combination with drug therapy highly limited the subgroup analysis and the comparison of therapeutic effects for herbal medicine combined with drug therapy to drug therapy alone. Such heterogeneity also makes it difficult to evaluate or identify herbal medicinal compounds effective for improving BD symptoms. Different types of integrative medicines and drug therapies used for different durations also made it difficult to compare the regimens. Fourth, herbal medicines used in the form of decoctions are not standardized and vary in their compositions and dosage, restricting the assessment of individual decoctions or single-herb effects. The diversity of herbal medicine administration within and across the included studies may have affected the overall results of our study, causing a certain degree of bias. Finally, we could not establish the safety of herbal medicine combined with drug therapy in treating BD, as almost half of the included studies did not report AEs.

### 4.4. Agreements and Disagreements with Other Studies or Reviews

This review showed that herbal medicine combined with drug therapy is potentially effective for symptom relief in BD patients. Two previous reviews studied the effect of herbal medicine combined with drug therapy in the treatment of BD [16,17]. In our review, we found nine additional RCTs that adhered to standard clinical diagnostic criteria. The results of the analysis showed that the symptom improvement rate was higher with herbal medicine combined with drug therapy than with drug therapy alone. This result is similar to that of previous reviews, showing that the inclusion of additional studies did not change the significance of the results. Despite the addition of updated evidence, the small sample size and poor quality of the included studies remain the major limitations of our review.

### 4.5. Implications for Clinical Practice

Clinical manifestations vary greatly among BD patients, and conventional therapy highly depends on the severity of the disease, which often involves several body systems and organs. The main principle of BD treatment via drug therapy remains to be subduing inflammation during attacks and improving patients’ quality of life by easing symptoms, increasing immunity, and reducing recurrence [40]. Although evidence supporting the use of azathioprine and cyclosporin A for ocular manifestations and interferon (IFN)α for mucocutaneous manifestations is available, evidence for vascular, gastrointestinal, and neurological involvement is still lacking [8]. To date, the effectiveness of drug therapy is based mostly on case reports/series and a limited number of RCTs.

Herbal medicine has been shown to have anti-inflammatory properties with favorable impacts on immune function and could play a critical role in complementing drug therapy [41]. The use of herbal medicine with anti-inflammatory properties also showed promising results in managing autoimmune diseases with oral manifestations, such as oral lichen planus, aphthous stomatitis, and Sjögren’s syndrome, revealing the increasingly important role of herbal medicine in managing various inflammatory diseases [42,43,44].

Although evidence for proving the effectiveness of herbal medicine combined with drug therapy in the treatment of BD remains weak, accumulating evidence still shows encouraging effects. Medical professionals may still cautiously recommend the use of herbal medicine combined with drug therapy to patients who present side effects and resistance to drug therapy over a long duration.

### 4.6. Implications for Research

There are several concerns regarding the use of herbal medicine combined with drug therapy for the treatment of BD. First, the herbal prescriptions and drug therapies used in the included studies varied across studies. The included studies might not have been sufficient to demonstrate the complete effects for treating BD, and a subgroup analysis was unable to be conducted due to the small number of studies included. Second, all studies had a short duration of treatment. BD is an autoimmune disease with a high rate of recurrence. It is necessary to prove the effectiveness of herbal medicine combined with drug therapy on the recurrence rate by extending the period of treatment. Third, a number of AEs were reported in the herbal medicine combined with drug therapy group, and this has raised concerns about possible herb–drug interactions, such as pharmacokinetic and pharmacodynamic (PK–PD) interactions. The integration of herbal medicines with conventional drugs may cause changes in the movement, absorption, biochemical, and physiological effects of the herbs. Herb–drug interactions due to the concurrent use of herbs with conventional drugs may also magnify the effects of drugs, plausibly leading to the effectiveness of herbal medicine combined with drug therapy in treating BD. Fourth, the herbs with the highest frequency of use across the studies were Glycyrrhizae Radix et Rhizoma, Scutellariae Radix, and Coptidis Rhizoma. [45,46,47] These herbs have shown great potential in the treatment of inflammation-related diseases due to their anti-inflammatory active compounds. Future studies on these herbs, as single herbs or decoctions, should be considered to validate their usage and effectiveness in treating BD. In general, well-designed RCTs in compliance with the CONSORT guidelines [48], long-term treatment periods, large sample sizes, and data on PK–PD parameters are warranted to guide the implementation of integrative medicine in clinical practice in the near future.

## 5. Conclusions

This review showed a significant improvement in symptoms upon the administration of herbal medicine combined with drug therapy in patients with BD. However, the included studies were performed on small sample sizes, had short treatment periods, and lacked detailed descriptions of the methodologies. To establish the effectiveness of herbal medicine combined with drug therapy in the treatment of BD, future RCTs designed in compliance with the CONSORT guidelines are required to ensure a larger sample size, longer treatment duration, and rigorous evidence-based treatment methodology.

## Figures and Tables

**Table 1:** Summary of randomized controlled trials on herbal medicine for the treatment of Behcet’s disease.

First Author (Year) [Ref]	Sample Size (Randomized/Analyzed) Mean Age/Disease Duration (Years) Diagnostic Criteria	Integrative Medicine (Regimen)	Drug Therapy (Regimen)	Main Outcomes	Results	Adverse Effect
Gong (2013) [23]	60/60 A: 36.5; B: 36.3/n.r. 1989 ISG; TCM diagnosis	(A) HM (Modified Gancao Xiexin decoction, 2 times daily for 3 months, *n* = 30), plus (B)	(B) Thalidomide (50 mg, once daily for 3 months, *n* = 30)	(1) Response rate (2) Recurrence rate (3) Symptom score (4) ESR (5) CRP	(1) RR 1.13 [0.89, 1.44], NS (2) 1 month: RR 0.33 [0.04, 3.03], NS; 2 months: RR 0.25 [0.06, 1.08], NS; 3 months: RR 0.27 [0.08, 0.88], *p* = 0.03 (3) Oral ulcer: MD 0.14 [−0.51, 0.79], NS; genital ulcer: MD 0.11 [−0.56, 0.78], NS; eye inflammation: MD −0.06 [−0.51, 0.39], NS; skin lesions: MD −0.44 [−0.83, −0.05], *p* = 0.03 (4) MD 0.10 [−3.91, 4.11], NS (5) MD −2.80 [−10.29, 4.69], NS	Dizziness and drowsiness (A: 1, B: 5); dry mouth and skin (A: 1, B: 3); foreign body sensation of skin (B: 1)
Zhang (2014a) [24]	60/60 A: 23.4; B: 24.8/A: 12.2; B: 12.6 TCM diagnosis	(A) HM (Modified Qingdai san, 2 times daily for 2 months, *n* = 30), plus (B)	(B) Thalidomide (50 mg, once daily for 2 months, *n* = 30)	(1) Response rate (2) Recurrence rate (3) ESR (4) CRP	(1) RR 1.32 [1.06, 1.65], *p* = 0.01 (2) 2 months: RR 0.25 [0.06, 1.08], NS (3) MD 0.23 [−2.91, 3.37], NS (4) MD −0.80 [−7.44, 5.84], NS	Multiple neuritis (B: 1)
Chen (2018) [25]	68/68 A: 38.3; B: 39.5/A: 3.8; B: 3.9 CRA	(A) HM (Shen’s Shengdi Qinlian Tufuling decoction, 2 times daily for 2 months, *n* = 34), plus (B)	(B) Thalidomide (25–50 mg, once daily for 2 months, *n* = 34)	(1) Response rate (2) ESR (3) CRP	(1) RR 1.10 [0.94, 1.30], NS (2) MD −6.10 [−11.79, −0.41], *p* = 0.04 (3) MD −1.80 [−6.80, 3.20], NS	Constipation (A: 1, B: 10); dizziness (A: 1, B: 4); limb numbness (B: 1); nausea (B: 1)
Huang (2018) [26]	60/60 A: 38.0; B: 41.3/A: 5.0; B: 4.8 2013 ICBD; 2002 GCR−TCM	(A) HM (Modified Huanglian Wendan decoction, 2 times daily), plus (B)	(B) Thalidomide (50 mg, 3 times daily; Celecoxib 0.2 g, 2 times daily for 3 months, *n* = 30)	(1) Response rate (2) Symptom score (3) ESR (4) CRP	(1) RR 1.17 [0.93, 1.48], NS (2) Oral ulcer: MD −0.63 [−1.09, −0.17], *p* = 0.007; genital ulcer: MD −0.73 [−1.15, −0.31], *p* = 0.0006; eye inflammation: MD −0.60 [−1.11, −0.09], *p* = 0.02; skin lesions: MD −0.66 [−1.15, −0.17], *p* = 0.009 (3) MD −2.23 [−4.07, −0.39], *p* = 0.02 (4) MD −2.25 [−4.16, −0.34], *p* = 0.02	None
Qu (2016) [27]	40/40 A: 37.0; B: 37.3/A: 3.5; B: 3.8 1989 ISG; TCM diagnosis	(A) HM (Modified Yiqi Jiedu Quyu prescription, 2 times daily for 3 months, *n* = 20), plus (B)	(B) Thalidomide (50 mg, once daily for 3 months, *n* = 20)	(1) Response rate (2) ESR (3) CRP	(1) RR 1.12 [0.91, 1.38], NS (2) MD −7.32 [−12.41, −2.23], *p* = 0.005 (3) MD −0.88 [−5.89, 4.13], NS	Constipation (B: 3); dizziness and drowsiness (A: 1, B: 2); hypomenorrhea (B: 1); pruritus (B: 1)
Liu (2015) [28]	46/43 A: 35.8; B: 35.7/A: 3.6; B: 3.6 1989 ISG	(A) HM (Huiyan Zhuyu decoction as tea substitute for 3 months, *n* = 22), plus (B)	(B) Thalidomide (75 mg, once daily for 3 months, *n* = 21)	Response rate	RR 1.43 [1.04, 1.96], *p* = 0.03	n.r.
Ma (2020) [29]	50/50 A: 37.3; B: 27.9/n.r. 1989 ISG	(A) HM (PI−based prescription for 3 months, *n* = 25), plus (B)	(B) Thalidomide (50~100 mg, once daily for 3 months, *n* = 25)	(1) Response rate (2) ESR (3) CRP	(1) RR 1.21 [0.94, 1.55], NS (2) Week 4: MD −1.00 [−4.35, 2.35], NS; week 8: MD −10.00 [−13.98, −6.02], *p* < 0.001; week 12: MD −12.00 [−16.61, −7.39], *p* < 0.001 (3) Week 4: MD −2.70 [−3.79, −1.61], *p* < 0.001; week 8: MD−1.40 [−2.54, −0.26], *p* = 0.02; week 12: MD −2.40 [−3.47, −1.33], *p* < 0.001	Dizziness (A: 2; B: 3); leukocytopenia (B: 1); liver damage (A: 1; B: 2); lower limb numbness (B: 1)
Liu (2008) [30]	100/100 A: 31; B: 32/A: 8.6; B: 8.4 1987 ISG	(A) HM (PI−based prescription, 2 times daily for 3 months, *n* = 60), plus (B)	(B) Prednisone (0.5~1 mg/kg, once daily for 3 months, *n* = 40)	Response rate	RR 1.21 [1.06, 1.38], *p* = 0.006	n.r.
Li (2011) [31]	60/60 A: 30.0; B: 31.0/A: 7.0; B: 8.0 1990 ISG	(A) HM (PI−based prescription, 2 times daily for 3 months, *n* = 30), plus (B)	(B) Prednisone (0.5~1 mg/kg, once daily for 3 months, *n* = 30)	Response rate	RR 1.33 [1.04, 1.72], *p* = 0.03	n.r.
Zhang (2014b) [32]	80/80 A: 30.0; B: 32.0/A: 7.0; B: 8.0 1990 ISG	(A) HM (Baitouweng decoction + Yinchenhao decoction, 2 times daily for 3 months, *n* = 40), plus (B)	(B) Prednisone (0.5~1 mg/kg, once daily for 3 months, *n* = 40)	Response rate	RR 1.04 [0.82, 1.32], NS	n.r.
Yan (2019) [33]	30/30 A: 30.0; B: 32.0/A: 7.9; B: 8.3 1990 ISG	(A) HM (Duanxia Shenshi decoction + Longdan Xiegan decoction, 2 times daily for 3 months, *n* = 30), plus (B)	(B) Prednisone (0.5~1 mg/kg, once daily for 3 months, *n* = 30)	Response rate	RR 1.33 [1.04, 1.72], *p* = 0.03	n.r.
Li (2016) [34]	114/114 A: 41.3; B: 41.3/n.r. ISG	(A) HM (Modified Chixiaodou Danggui San, 2 times daily for 2 weeks, *n* = 57), plus (B)	(B) Prednisone (20~60 mg, once daily; Levamisole 50 mg, 3 times daily for 2 weeks, *n* = 57)	Response rate	RR 1.20 [1.04, 1.39], *p* = 0.02	n.r.
Zhu (2011) [35]	67/67 A: 32.5; B: 30.0/n.r. 1989 ISG	(A) HM (Modified Ziyin Yuyang decoction, 2 times daily for 2 months, *n* = 37), plus (B)	(B) Prednisone (1 mg/kg once daily) + Cyclophosphamide 200 mg, 3 times weekly for 2 months, *n* = 30)	Response rate	RR 1.20 [0.96, 1.49], NS	n.r.
Wang (2005) [36]	42/42 A: 34.2; B: 38.7/A: 3.6; B: 3 1989 ISG	(A) HM (Leiling Jiedu decoction, 2 times daily for 2 months, *n* = 22), plus (B)	(B) Prednisone (30~40 mg, once daily for 2 months) + Dexamethasone (10 mg, once daily for the 1st week, *n* = 20)	Response rate	RR 1.25 [0.99, 1.57], NS	None
Chang (2020) [37]	60/60 A: 31.4; B: 32.07/A: 2.48; B: 2.51 1989 ISG	(A) HM (Modified Huatan Quyu prescription, 2 times daily for 2 months, *n* = 40), plus (B)	(B) Azathioprine (100~150 mg, once daily for 2 months, *n* = 20)	(1) Response rate (2) ESR (3) CRP	(1) RR 1.12 [0.92, 1.36], NS (2) Reported only as *p* < 0.05 (3) Reported only as *p* < 0.05	Leukocytopenia (A: 3; B: 4); liver damage (A: 5; B: 9); nausea and vomiting (A: 2; B: 3); skin rashes (A: 2; B: 2)
Gu (2015) [38]	50/50 A: 30.2; B: 29.6/A: 2.5; B: 2.3 CRA, TCM diagnosis	(A) HM (Modified Huatan Quyu prescription, 2 times daily for 2 months, *n* = 27), plus (B)	(B) Azathioprine (100 mg, once daily for 2 months, *n* = 23)	(1) Response rate (2) CRP	(1) RR 1.11 [0.93, 1.32], NS (2) MD −2.87 [−4.64, −1.10], *p* = 0.002	Constipation (A: 1); dizziness and headache (B: 2); drowsiness (A: 4); edema (A: 1); leukocytopenia (A: 1, B: 1); loss of appetite (B: 2); nausea and vomiting (B: 3); peripheral sensory neuropathy (A: 2); skin rashes (A: 1, B: 1)

CRA: Chinese Rheumatology Association; CRP: C-reactive protein; ESR: erythrocyte sedimentation rate; GCR-TCM: Guiding principle of clinical research on new drugs of traditional Chinese medicine; HM: herbal medicine; ICBD: International Criteria for Behcet’s disease; ISG: International Study Group Criteria; n.r.: not reported; NS: nonsignificant; PI: pattern identification; MD: mean difference; RR: risk ratio; TCM: traditional Chinese medicine.

**Table 2:** Compositions of the herbal medicines for the treatment of Behcet’s disease.

Author (Year) [Ref]	Prescription	Composition of Herbs
Gong (2013) [23]	Modified Gancao Xiexin decoction	Glycyrrhizae Radix et Rhizoma 9 g, Glycyrrhizae Radix et Rhizoma Praeparata 9 g, Scutellariae Radix 9 g, Coptidis Rhizoma 6 g, Angelicae Gigantis Radix 15 g, Astragali Radix 18 g, Coicis Semen 30 g, Zingiberis Rhizoma 6 g, Paeoniae Radix Rubra 9 g, Moutan Cortex 9 g, Lonicerae Flos 20 g, Forsythiae Fructus 9 g, Citri Pericarpium 6 g Modification based on symptoms: [amenorrhea, forgetfulness, insomnia: Ligustri lucidi Fructus 20 g, Polygoni Multiflori Ramulus 20 g]; [nausea, vomiting, loss of appetite: Atractylodis Macrocephalae Rhizoma 15 g, Amomi Fructus 6 g]; [vexing sensation in chest, palms and soles, tidal fever: Anemarrhenae Rhizoma 12 g, Phellodendri Cortex 6 g]; [night sweating or osteopyrexia: Lycii Radicis Cortex12 g]
Zhang (2014a) [24]	Modified Qingdai san, topical administration	Indigo Naturalis, Coptidis Rhizoma, Phellodendri Cortex, Natrii Sulfas, Bomeolum
Chen (2018) [25]	Shen’s Shengdi Qinlian Tufuling decoction	Rehmanniae Radix 30 g, Scutellariae Radix 30 g, Coptidis Rhizoma 6 g, Smilacis Glabrae Rhizoma 30 g, Caraganae Radix 30 g, Zedoariae Rhizoma 30 g, Paeoniae Radix Rubra 15 g, Moutan Cortex 15 g
Huang (2018) [26]	Modified Huanglian Wendan decoction	Pinelliae Rhizoma 25 g, Coptidis Rhizoma 20 g, Bupleuri Radix 10 g, Citri Pericarpium 15 g, Atractylodis Macrocephalae Rhizoma 20 g, Lonicerae Flos 15 g, Smilacis Glabrae Rhizoma 20 g, Plantaginis Semen 10 g, Moutan Cortex 15 g, Glycyrrhizae Radix et Rhizoma 10 g
Qu (2016) [27]	Modified Yiqi Jiedu Quyu prescription	Astragali Radix 30 g, Rehmanniae Radix 30 g, Zedoariae Rhizoma 15 g, Scutellariae Radix 30 g, Smilacis Glabrae Rhizoma 30 g, Caraganae Radix 30 g, Rhei Radix et Rhizoma 9 g, Glycyrrhizae Radix et Rhizoma 12 g, Glycyrrhizae Radix et Rhizoma Praeparata 12 g Modification based on symptoms: [erythema in the legs: Ranunculi Tuber 15 g, Forsythiae Fructus 12 g]; [vulvar ulcer: Millettiae Caulis 30 g, Sophorae Radix 15 g]; [redness and pain in eyes: Bupleuri Radix 9 g, Gardeniae Fructus 9 g]; [bitter taste in the mouth: Coptidis Rhizoma 9 g, Lophatheri Herba 9 g]; [dry mouth, mild fever: Anemarrhenae Rhizoma 9 g, Phellodendri Cortex 9 g]
Liu (2015) [28]	Huiyan Zhuyu decoction, as tea substitute	Rehmanniae Radix 50 g, Scrophulariae Radix 50 g, Aurantii Fructus 50 g, Persicae Semen 30 g, Angelicae Gigantis Radix 50 g, Carthami Flos 30 g, Bupleuri Radix 30 g, Paeoniae Radix Rubra 30 g, Platycodi Radix 30 g, Glycyrrhizae Radix et Rhizoma 30 g Modification based on PI: [dampness-heat pattern: Taraxaci Herba 20 g, Moutan Cortex 30 g]; [heat toxin pattern: Coicis Semen 50 g, Scutellariae Radix 30 g]; [yin deficiency pattern: Anemarrhenae Rhizoma 50 g, Junci Medulla 10 g, Ecliptae Herba 30 g]; [spleen–stomach yin deficiency pattern: Astragali Radix 50 g, Codonopsis Pilosulae Radix 30 g]
Ma (2020) [29]	(1) Modified Wuwei Xiaodu Yin (syndrome of retained dampness toxin) (2) Gancao Xiexin decoction (syndrome of retained dampness-heat) (3) Baihe Dihuang decoction/Modified Zhibai Dihuang decoction (syndrome of yin deficiency with inner heat)	(1) Lonicerae Flos 20 g, Chrysanthemi Flos 20 g, Violae Herba 20 g, Moutan Cortex 15 g, Gentianae Radix 15 g, Bupleuri Radix 15 g, Poria Sclerotium 20 g, Coicis Semen 20 g (2) Glycyrrhizae Radix et Rhizoma 10 g, Scutellariae Radix 15 g, Zingiberis Rhizoma 10 g, Coptidis Rhizoma 10 g, Pinelliae Praeparatum cum Zingiberis 20 g, Zizyphi Fructus 3 pieces (3) Anemarrhenae Rhizoma 20 g, Phellodendron chinense Schneid. 15 g, Lilii Bulbus 20 g, Rehmanniae Radix 20 g, Poria Sclerotium 20 g, Chrysanthemi Flos 20 g, Moutan Cortex 20 g Modification based on symptoms: [swelling and pain in joints: Millettiae Caulis 20 g, Gentiana macrophylla Pallas 20 g, Achyranthes japonica Nakai 20 g, Sinomenium acutum 10 g]; [erythema nodosum: Manis pentadactyla 15 g, Melandrium firmum Rohrbach 15 g, Paeoniae Radix Rubra 20 g]; [redness and blurriness in eyes: Celosia argentea 15 g, Buddleja officinalis Maximowicz 15 g, Gardeniae Fructus 20 g]; [dry stools: Rhei Radix et Rhizoma 8 g, Magnoliae Cortex 15 g]; [insomnia: Polygonum multiflorum 20 g, Ziziphus jujuba Mill.var.spinosa 20 g]
Liu (2008) [30]	(1) Xiegan san (syndrome of retained dampness-heat toxin) (2) Danggui Liuhuang decoction (syndrome of yin deficiency with heat toxin) (3) Renshen Maidong san (syndrome of dual deficiency of qi and blood)	(1) Scrophulariae Radix 10 g, Rhei Radix et Rhizoma 10 g, Scutellariae Radix 10 g, Platycodi Radix 10 g, Angelicae Gigantis Radix 10 g, Natrii Sulfas 10 g, Gentianae Radix 10 g, Plantaginis Semen 15 g, Notopterygii Rhizoma 6 g, Anemarrhenae Rhizoma 12 g (2) Angelicae Gigantis Radix 10 g, Rehmanniae Radix 10 g, Rehmanniae Radix Praeparata 10 g, Coptidis Rhizoma 10 g, Scutellariae Radix 10 g, Phellodendri Cortex 10 g, Astragali Radix 20 g (3) Ginseng Radix 10 g, Atractylodis Rhizoma 10 g, Scutellariae Radix 10 g, Anemarrhenae Rhizoma 10 g, Glycyrrhizae Radix et Rhizoma Praeparata 10 g, Bambusae Caulis in Taeniam 10 g, Rehmanniae Radix 12 g, Liriopis Tuber 20 g
Li (2011) [31]	(1) Longgan Xiegan decoction (syndrome of retained dampness heat toxin) (2) Zhibai dihuang decoction (syndrome of yin deficiency with heat toxin) (3) Modified Shengmai yin (syndrome of dual deficiency of qi and blood)	(1) Gentianae Radix 12 g, Scutellariae Radix 12 g, Rehmanniae Radix 12 g, Bupleuri Radix 10 g, Bupleuri Radix 10 g, Alismatis Rhizoma 10 g, Plantaginis Semen 10 g, Angelicae Gigantis Radix 10 g, Akebiae Caulis 6 g, Glycyrrhizae Radix et Rhizoma Praeparata 6 g (2) Anemarrhenae Rhizoma 12 g, Phellodendri Cortex 12g, Rehmanniae Radix Praeparata 12 g, Moutan Cortex 12 g, Dioscoreae Rhizoma 12 g, Corni Fructus 10 g, Atractylodis Rhizoma 10 g, Alismatis Rhizoma 10 g (3) Panacis Quinquefolii Radix 6 g, Schizandrae Fructus 6 g, Glycyrrhizae Radix et Rhizoma Praeparata 6 g, Liriopis Tuber 20 g, Astragali Radix 20 g, Ecliptae Herba 20 g
Zhang (2014b) [32]	Baitouweng decoction + Yinchenhao decoction	Pulsatillae Radix 12 g, Coptidis Rhizoma 9 g, Phellodendri Cortex 9 g, Fraxini Cortex9 g, Artemisiae Scopariae Herba 30 g, Rhei Radix et Rhizoma9 g, Gardeniae Fructus 9 g
Yan (2019) [33]	Duanxia Shenshi decoction + Longdan Xiegan decoction	Atractylodis Rhizoma 6 g, Phellodendri Cortex 9 g, Polyporus 9 g, Atractylodis Rhizoma 9 g, Crataegii Fructus 9 g, Lonicerae Flos 12 g, Ailanthus Altissima Swingle 12 g, Sanguisorbae Radix 9 g, Gentianae Radix 9 g, Angelicae Gigantis Radix 9 g, Gardeniae Fructus 9 g, Akebiae Caulis 6 g, Plantaginis Semen 6 g, Plantaginis Semen 6g, Scutellariae Radix 9 g, Glycyrrhizae Radix et Rhizoma 6 g, Rehmanniae Radix 9 g, Alismatis Rhizoma 9 g
Li (2016) [34]	Modified Chixiaodou Danggui San	Phaseoli Semen 15 g, Angelicae Gigantis Radix 15 g, Scutellariae Radix 6 g, Coptidis Rhizoma 6 g, Sophorae Radix 6 g, Plantaginis Semen 6 g, Akebiae Caulis 6 g, Phyllostachys Folium 6 g, Glycyrrhizae Radix et Rhizoma 6 g
Zhu (2011) [35]	Modified Ziyin Yuyang decoction	Rehmanniae Radix Praeparata 15 g, Angelicae Gigantis Radix 15 g, Anemarrhenae Rhizoma 10 g, Liriopis Tuber 10 g, Phellodendri Cortex 10 g, Cuscutae Semen 9 g, Ligustri Lucidi Fructus 9 g, Paeoniae Radix Alba 9 g, Moutan Cortex 9 g, Cinnamomi Cortex 6 g, Glycyrrhizae Radix et Rhizoma 6 g Modification based on PI: [dampness-heat pattern: Atractylodis Rhizoma 10 g; qi deficiency pattern: Astragali Radix 15 g]
Wang (2005) [36]	Leiling Jiedu decoction	Smilacis Glabrae Rhizoma 15 g, Codonopsis Pilosulae Radix 15 g, Tripterygii Cortex 10 g, Angelicae Gigantis Radix 15 g, Salviae Miltiorrhizae Radix 10 g, Lithospermi Radix 15 g, Rehmanniae Radix 15 g, Oldenlandiae Diffusae Herba 15 g, Glycyrrhizae Radix et Rhizoma 10 g Modification based on PI: [liver–kidney yin deficiency pattern: Rehmanniae Radix Praeparata 10 g, Scrophulariae Radix 10 g, Lycii Fructus 15 g]; [spleen–kidney yang deficiency pattern: Aconiti Iateralis Radix Praeparata 10 g, Cinnamomi Ramulus 10 g]; [dual deficiency of qi and blood pattern: Astragali Radix 10 g, Atractylodis Macrocephalae Rhizoma 10 g]
Chang (2020) [37]	Modified Huatan Quyu prescription	Arisaema amurense Maximowicz var. serratum Nakai 9 g, Curcuma phaeocaulis Valeton 10 g, Brassicae Semen 10 g, Pinelliae Rhizoma 12 g, Angelicae Sinensis Radix 12 g, Persicae Semen 9g, Boswellia carterii Birdwood 10 g, Commiphora myrrha 9 g, Zingiberis Rhizoma Recens 15 g, Chuanxiong Rhizoma 6 g, Glycyrrhizae Radix et Rhizoma 30 g, Prunella vulgaris Linné 12 g, Notopterygii Rhizoma seu Radix 15 g
Gu (2015) [38]	Modified Huatan Quyu prescription	Pinelliae Rhizoma 15 g, Angelicae Gigantis Radix 9 g, Rehmanniae Radix 9 g, Atractylodis Rhizoma 9 g, Persicae Semen 12 g, Zingiberis Rhizoma Recens 25 g, Paeoniae Radix Rubra 9 g, Cnidii Rhizoma 6 g, Citri Pericarpium 15 g, Glycyrrhizae Radix et Rhizoma 30 g, Commelinae Herba 12 g, Carpesii Fructus 15 g, Euonymi Lignum Suberalatum 12 g, Zaocys Praeparata 12 g Modification based on PI: [liver dampness-heat pattern: Gentianae Radix, Phellodendri Cortex, Phaseoli Semen] Modification based on symptoms: [vulvar ulcer: Kochiae Fructus]; [erosions of anus: Sophorae Fructus Praeparata]; [eye damage: Buddlejae Flos, Cassiae Semen]; [mouth ulcer (external): Bomeolum, Borax, Cinnabaris, Natrii Sulfas Exsiccatus]

**Table 3:** Summary of Findings.

Integrative Medicine Compared to Drug Therapy for Behcet’s Disease					
**Patient or population:** Patients with Behcet’s disease **Setting:** Hospital outpatients (Study design: randomized controlled trial) **Intervention:** Integrative medicine **Comparison:** Drug therapy					
**Outcome**	**№ of Participants** **(Studies)** **Follow-Up**	**Certainty of Evidence**	Relative Effect * **(95% CI)**	**Anticipated Absolute Effects**	
				**Risk with Drug Therapy**	**Risk Difference with Integrative Medicine**
Response rate	1034 (16 RCTs)	⨁⨁◯◯ LOW ^a^^,^^b^	RR 1.19 (1.13 to 1.25)	776 per 1000	**147 more per 1000** (101 more to 194 more)
Recurrence rate (2 months)	120 (2 RCTs)	⨁⨁◯◯ LOW ^a^^,^^b^	RR 0.27 (0.09 to 0.76)	250 per 1000	**183 fewer per 1000** (228 fewer to 60 fewer)
Oral ulcers	120 (2 RCTs)	⨁◯◯◯ VERY LOW ^a^^,^^b^^,^^c^	-		MD **0.28 lower** (1.03 lower to 0.47 higher)
Genital ulcers	120 (2 RCTs)	⨁◯◯◯ VERY LOW ^a^^,^^b^^,^^c^	-		MD **0.35 lower** (1.17 lower to 0.47 higher)
Eye inflammation	120 (2 RCTs)	⨁◯◯◯ VERY LOW ^a^^,^^b^^,^^c^	-		MD **0.32 lower** (0.84 lower to 0.21 higher)
Skin lesions	120 (2 RCTs)	⨁⨁◯◯ LOW ^a^^,^^b^	-		MD **0.52 lower** (0.83 lower to 0.22 lower)
ESR	338 (6 RCTs)	⨁◯◯◯ VERY LOW ^a^^,^^b^^,^^c^	-		MD **4.19 lower** (7.59 lower to 0.79 lower)
CRP	388 (7 RCTs)	⨁⨁◯◯ LOW ^a^^,^^b^	-		MD **2.4 lower** (3.19 lower to 1.6 lower)

* The risk in the intervention group (and its 95% confidence interval) is based on the assumed risk in the comparison group and the relative effect of the intervention (and its 95% CI). CI: confidence interval; MD: mean difference; RCT: randomized controlled trial; RR: risk ratio; ^a^. Overall risk of bias is uncertain. Only four studies reported the simple randomization method, and the remaining studies did not provide relevant information. All studies reported a lack of allocation concealment and blinding. Other risk of bias domains were also concerning due to poor reporting. Therefore, the studies included were judged to have serious methodological limitations. ^b^. The sample size of each study is considered small, resulting in borderline imprecision. ^c^. Heterogeneity across the studies is fairly high. GRADE Working Group grades of evidence. High certainty: We are very confident that the true effect lies close to that of the estimate of the effect. Moderate certainty: We are moderately confident in the effect estimate: the true effect is likely to be close to the estimate of the effect, but there is a possibility that it is substantially different. Low certainty: Our confidence in the effect estimate is limited: the true effect may be substantially different from the estimate of the effect. Very low certainty: We have very little confidence in the effect estimate: the true effect is likely to be substantially different from the estimate of effect.

## Data Availability

Data sharing not applicable.

## Supplementary Materials

The following are available online at https://www.mdpi.com/article/10.3390/pharmaceutics13040476/s1.
