# Supplementary material for: Integrative Medicine (Herbal Medicine Combined with Drug Therapy) for Behcet’s Disease: A Systematic Review and Meta-Analysis of Randomized Controlled Trials
Source: Pharmaceutics. 2021 Apr 1;13(4):476. doi: 10.3390/pharmaceutics13040476 (PMC8065893; doi:10.3390/pharmaceutics13040476)
Supplement: Supplementary file 1 [file pharmaceutics-13-00476-s001.pdf]

pharmaceutics

Supplement Materials: Integrative Medicine (Herbal Medicine Combined with Drug Therapy) for Behcet's Disease: A Systematic Review and Meta-Analysis of Randomized Controlled Trials

Ji Hee Jun, Lin Ang, Tae Young Choi, Hye Won Lee and Myeong Soo Lee

Search strategies

1. PubMed

#1. "behcet syndrome" [mesh]
#2. "Behcet's disease" or "Behcet syndrome" or "Adamantiades-Behcet's Syndrome" or "Hulusi-behcet's syndrome" or "Ouclo-bucco-genital syndrome" or "Touraine's aphthosis" or "Triple Symptom Complex of Behcet" [tw]
#3. "Behcet's disease" or "Behcet syndrome" or "Adamantiades-Behcet's Syndrome" or "Hulusi-behcet's syndrome" or "Ouclo-bucco-genital syndrome" or "Touraine's aphthosis" or "Triple Symptom Complex of Behcet" [tiab]
#4. BD. ab.
#5. OR/ 1-4
#6. exp Medicine, Herbal/
#7. exp Plants, Medicinal/
#8. exp Medicine, Traditional/
#9. exp Drugs, Chinese Herbal/
#10. exp drugs, non-prescription/
#11. exp complementary therapies/
#12. exp Integrative Medicine
#13. ((traditional or chinese or herbal) adj (medic$ or preparation$ or drug$)).tw
#14. ((oriental or chinese) adj tradition$).tw.
#15. ((alternative or complementary) adj (therap$ or medicine$)).tw.
#16. OR/ 6-15
#17. 5 AND 16

2. EMBASE

#1. exp Behcet syndrome /
#2. "Behcet's disease" or "Behcet syndrome" or "Adamantiades-Behcet's Syndrome" or "Hulusi-behcet's syndrome" or "Ouclo-bucco-genital syndrome" or "Touraine's aphthosis" or "Triple Symptom Complex of Behcet". tw.
#3. BD. ab.
#4. OR/1-3
#5. exp Medicine, Herbal/
#6. exp Plants, Medicinal/
#7. exp Medicine, Traditional/
#8. exp Drugs, Chinese Herbal/
#9. exp drugs, non-prescription/
#10. exp complementary therapies/
#11. ((traditional or chinese or herbal) adj (medic$ or preparation$ or drug$)).tw
#12. ((oriental or chinese) adj tradition$).tw.
#13. ((alternative or complementary) adj (therap$ or medicine$)).tw.
#14. exp Integrative Medicine
#15. OR/5-14
#16. 4 AND 15

3. Cochrane library

#1. "Behcet syndrome" [Mesh].

#2. “Behcet’s disease” or “Behcet syndrome” or “Adamantiades-Behcet’s Syndrome” or “Hulusi-behcet’s syndrome” or “Ouclo-bucco-genital syndrome” or “Touraine’s aphthosis” or “Triple Symptom Complex of Behcet”: ti, ab, kw

#3. BD. ab.

#4 OR/1-3

#5. “Medicine, Chinese Traditional” [Mesh]

#6. “Medicine, Kampo”[Mesh]

#7. “Medicine, Korean Traditional”[Mesh]

#8. “Complementary therapies” [Mesh]

#9. Herbal, Decoction or herbal medicine or traditional medicine or TCM or tang: ti, ab, kw

#10. alternative or complementary: ti, ab, kw

#9 OR/5-8

#10 4 AND 9

4. CNKI, VIP, Wanfang

#1. 白塞氏病

#2. 狐惑病

#3. 白塞氏综合征

#4. 白塞综合征

#5. 白塞病

#6. 贝赫切特病

#7. 贝赫切特综合征

#8. 眼-口-生殖器三联综合征

#9. 皮肤－黏膜－眼综合征

#10. Behcet syndrome

#11. Behcet’s syndrome

#12. Behcet’s disease

#13. BD

#14. beh-CHETS

#15. Adamantiades-Behcet’s Syndrome

#16. Hulusi-behcet’s syndrome

#17. Touraine’s aphthosis

#18.Triple Symptom Complex of Behcet

#19. OR/1-18

#20. 中药

#21. 中医

#22. 汤

#23. 中医药

#24. 方剂

#25. 方

#26. 中西结合

#27. 中西医

#28. Integrative medicine

#29. Chinese integrative medicine

#30. Herbal medicine

#31. Traditional Chinese medicine

#32. Traditional medicine
#33. Decoction
#34. TCM
#35. tang
#36. OR/20-35
#37. 19 AND 36

5. Korean databases (OASIS, DBpia, RISS, KISS, and Korea Med)

#1. 베체트병
#2. 아다만티아데스-배채트 증후군
#3. 베체트 증후군
#4. 후루시-베체트 증후군
#5. 눈-볼-생식기 증후군
#6. 우렌 아프타증
#7. 베체트 삼중 복합 증후군
#8. Behcet
#9. OR/1-8
#10. 한약
#11. 처방
#12. 탕
#13. 통합의학
#14. 중서결합
#15. herbal
#16. decoction
#17. traditional medicine
#18. Integrative medicine
#19. OR/10-18
#20. 9 AND 20
